# Emerging and Underdetected Colonization Factors in Enterotoxigenic *Escherichia coli* (ETEC)

**DOI:** 10.3390/microorganisms14092123

**Published:** 2026-09-21

**Authors:** Astrid von Mentzer

**Affiliations:** 1 Department of Microbiology and Immunology, Institute of Biomedicine, Sahlgrenska Academy, University of Gothenburg, 405 30 Gothenburg, Sweden; astrid.von.mentzer@gu.se; 2 Wallenberg Centre for Molecular Translation Medicine, University of Gothenburg, 405 30 Gothenburg, Sweden; 3 SciLifeLab, University of Gothenburg, 405 30 Gothenburg, Sweden

**Keywords:** ETEC, colonization factors, detection, genomics, diagnostics, diarrhea

## Abstract

Adhesionto the gut epithelium is the first step in ETEC infection and is mediated by colonization factors (CFs). CFs are also the main targets of the ETEC vaccines currently in development, which are based on a small set of CFs considered to be the most prevalent. However, a large proportion of clinical isolates are reported as CF-negative when screened with standard PCR panels. This is partly because ETEC adhesins are more diverse than previously appreciated, and partly because the methods used to detect them only screen for a small, historically defined set of CFs. These two explanations are difficult to separate, and both suggest that CF diversity, and with it the coverage of current CF-based vaccines, has likely been underestimated. This review examines emerging and underdetected CFs in human ETEC, focusing on κ- and γ-clade adhesins such as CS23 and their links to animal-associated fimbriae. It discusses the implications of this growing diversity for vaccine development, the need for continued surveillance as the CF landscape evolves, and approaches to achieving broader vaccine coverage across the expanding diversity of ETEC adhesins.

## 1. Introduction

Enterotoxigenic Escherichia coli (ETEC) is a major cause of diarrhea across all age groups, although children below five years of age are a particularly vulnerable group. ETEC colonizes the small intestine and delivers heat-labile and/or heat-stable toxins, and adhesion is the prerequisite step involving so-called colonization factors (CFs).

The true burden of ETEC is difficult to assess, but the pathotype is estimated to cause 220 million diarrheal cases each year globally [1,2]. Large epidemiological studies and meta-analyses in low- and middle-income countries further highlight ETEC as a leading bacterial cause of diarrhea in young children, with substantial variation in prevalence across regions and studies [3]. In travelers, ETEC is recognized as the most common bacterial pathogen associated with travelers’ diarrhea (TD), particularly among individuals visiting endemic regions with inadequate water sanitation and hygiene infrastructure [4,5].

The overall mortality associated with ETEC infections has declined over recent decades. However, this reduction likely underestimates the true burden, as it does not fully capture the long-term consequences of repeated diarrheal episodes in early childhood. These include increased susceptibility to recurrent enteric infections, intestinal inflammation, malnutrition, micronutrient deficiencies, growth faltering, and impaired cognitive development, which are more difficult to quantify but remain critically important contributors to disease burden [6].

ETEC not only impacts human health but is also a major pathogen in the livestock industry, where it causes diarrheal disease in young animals, including piglets, calves, and lambs [7]. These infections are associated with significant economic losses and have a negative impact on animal welfare [7].

Although antimicrobial resistance is not a defining feature of ETEC, increasing resistance in *Escherichia coli* (*E. coli*) more broadly, together with reports of resistance in ETEC isolates, highlights the importance of considering these pathogens within a One Health framework [8,9,10,11,12,13]. The widespread use of antibiotics for diarrheal disease may further contribute to the emergence and dissemination of antimicrobial resistance in ETEC and related enteric pathogens [10,14].

A key step in ETEC pathogenesis is adherence to the epithelial cells of the small intestine, mediated by CFs [15]. These adhesins are essential virulence determinants and constitute the primary targets of current ETEC vaccine candidates [16,17,18,19]. CFs included in current ETEC vaccine formulations (for an extended list see von Mentzer and Svennerholm [15]) are selected based on their reported prevalence [20] and are intended to provide broad coverage of circulating ETEC strains by targeting the most frequently detected colonization factors. Notably, a substantial proportion of ETEC isolates—often ranging from 30% to 50% depending on the study and geographic region—are reported as CF-negative when screened using standard PCR panels targeting the most common CFs. This raises the possibility that many isolates classified as CF-negative may instead express less well-characterized or previously unrecognized CFs that are not included in routine diagnostics [15]. A central theme of this review is that the diversity of ETEC CFs is shaped simultaneously by real evolutionary diversification and by the limitations of the methods used to detect it, and that these two forces are difficult to disentangle: strains carrying divergent or rarely screened adhesins are both biologically distinct and, by construction, invisible to conventional surveillance.

Recent genomic studies have considerably expanded our understanding of the diversity of colonization factors (CFs) in ETEC. However, interpreting these findings is not always straightforward, as an increased detection of a CF may reflect either a genuine change in the epidemiology of ETEC or simply improvements in our ability to detect previously overlooked adhesins. Throughout this review, we therefore distinguish between two concepts that help interpret these observations: *emerging* and *underdetected* colonization factors.

In this review, we consider a CF to be *emerging* when there is evidence that its epidemiological importance has increased over time. This may be reflected by increasing prevalence in longitudinal surveillance studies, detection in multiple contemporary collections, expansion into new geographic regions or host populations, or dissemination across multiple ETEC lineages. Importantly, demonstrating emergence requires representative surveillance over time, as increased detection alone is not sufficient evidence that a CF is becoming more common.

In contrast, a CF is considered *underdetected* when available evidence suggests that its reported prevalence has been underestimated because of methodological limitations rather than true biological rarity. Historically, most epidemiological studies have relied on PCR panels targeting a limited number of well-characterized CFs. Consequently, CFs absent from these panels, or exhibiting substantial sequence variation that compromises primer binding, would not have been detected regardless of their prevalence in the population. The increasing use of whole-genome sequencing and expanded molecular screening has revealed that several CFs previously considered uncommon are likely to be more widespread than originally appreciated.

Importantly, these concepts are not mutually exclusive. A CF may be both emerging and historically underdetected. For example, improved genomic surveillance may first reveal that a CF has been consistently overlooked, while longitudinal data subsequently demonstrate that its prevalence is also increasing. Distinguishing between these two processes is important when interpreting surveillance studies, assessing vaccine coverage, and understanding the ongoing evolution of ETEC colonization factors.

Finally, the epidemiological status of a CF should be considered separately from its degree of experimental characterization. Identification of a CF operon by genome sequencing demonstrates its genetic presence but does not establish biological function. Experimental evidence, including demonstration of fimbrial expression, epithelial adherence, receptor binding, mutagenesis, or animal infection studies, provides increasing confidence that a predicted CF contributes to colonization. Consequently, a CF may be emerging, underdetected, and either well characterized or remain largely uncharacterized experimentally.

In this review, we focus on emerging and underdetected CFs in human ETEC, their diversity and evolutionary relationships including links to animal-associated fimbriae, and how detection limitations affect surveillance and vaccine design.

## 2. Colonization Factors in ETEC-Diversity and Biological Context

### 2.1. Diversity of CFs in Human and Animal ETEC

To date, 29 colonization factors (CFs) have been described in human ETEC isolates, reflecting the extensive heterogeneity of this pathotype. The currently described human CFs have recently been comprehensively reviewed [15]. Human ETEC isolates frequently express multiple CFs, with common combinations including CFA/I+CS21, CS1+CS3+CS21, and CS2+CS3+CS21 [15,20]. CS6 is unusual, compared to other CFs, because it is frequently co-harbored with distinct CFs, including CS4, CS5, CS8 and CS21 [21]. The presence of multiple CFs complicates the attribution of individual CFs to specific functional roles in colonization and pathogenesis.

Many CFs are structurally and genetically heterogeneous and can be grouped into related families, particularly the class 5 fimbriae family, which includes CFA/I, CS1, CS2, CS4, CS14, and CS17. In addition, the global distribution of CFs varies considerably, with certain CFs predominating in specific geographic regions, age groups, and host populations [20,22,23,24,25,26]. A degree of heterogeneity is observed within individual CFs. CFA/I appear relatively conserved both genetically and antigenically, others, such as CS6, exhibit considerable variation [27] with multiple alleles of major subunits reported as well as being identified in multiple plasmid backgrounds and phylogenetic lineages [22,24,27,28].

In contrast, animal-associated ETEC express a more limited number of CFs—F4, F5, F6, F17, F18, and F41—most often expressed as the sole CF. These CFs account for the majority of strains causing disease in livestock [15].

### 2.2. Structural Classes of CFs

The majority of ETEC CFs are assembled by the chaperone–usher (CU) pathway, in which periplasmic chaperones escort subunit proteins to an outer-membrane usher that catalyzes their polymerization into the mature surface structure [29]. Within this pathway, the classical Class 5 fimbriae (the CFA/I-like CFs and CS5/CS7) are assembled via a distinct, alternate chaperone–usher (ACU) system, whereas other CFs use the classical CU pathway (Table 1). CU-assembled CFs adopt a range of morphologies—rigid fimbriae (e.g., CFA/I, CS1), thinner fibrillae (e.g., CS3), double-helical fibril (CS5, CS7), and afimbrial or non-fimbrial structures (e.g., CS6)—conventionally distinguished by electron microscopy and, increasingly, predicted from sequence (Table 1) [15]. Despite this morphological variation, these CFs are assembled in a similar manner and also share a common operon organization with core functional units—major subunit, minor subunit(s), chaperone and usher—occasionally including a tip adhesin that determines receptor specificity. ETEC may also express type IV pili, specifically CS21 (Longus) and CS8 (CFA/III), which are assembled by a type IV machinery forming retractile filaments implicated in adhesion, microcolony formation and biofilm development [30]. Finally, ETEC encode several non-classical adhesins that act independently of the CU machinery, including the two-partner secretion exoprotein EtpA and autotransporter or serine-protease adhesins such as EatA, which mediate host-cell binding directly and are conserved across diverse ETEC and other *E. coli* [31,32,33]. Since, these non-canonical virulence factors are shared broadly across strains, they have been highlighted as potential broad-coverage vaccine targets (Section 6). Recent genomic analyses have further identified additional CU-pilus subclasses and cryptic adhesin loci in strains that screen negative for known CFs [34,35], indicating that the structural repertoire of ETEC CFs is broader than the current set implies—a point developed for specific emerging CFs in Section 4.

### 2.3. Evolutionary Classification of CFs

Based on sequence similarity of the conserved outer membrane usher proteins, CU-assembled CFs can be grouped into distinct fimbrial usher protein (FUP) clades [29], primarily including the α-, γ-, and κ-clades. The α-clade comprises the classical CFA/I-like CFs, which are among the most prevalent and best characterized. The γ-clade includes several structurally and genetically diverse CFs, including both common and less prevalent types, and can be further subdivided into multiple subgroups including γ2-FUP (CS12, CS18, CS20, CS26, CS30, F6), γ3-FUP (CS3, CS6) and γ4-FUP (F17a).

In contrast, the κ-FUP clade contains CFs that are more closely related to animal-associated CFs. Notably, both γ2-FUP and κ-FUP include CFs identified in both human and animal isolates, pointing to evolutionary links between human and animal ETEC that are discussed in Section 5.

CFs are also commonly grouped into CFA/I-like, CS5-like, and Class 1b families. These two schemes have different origins and are not interchangeable. The family classification predates routine sequencing and was built on shared antigenic properties and serological cross-reactivity, whereas the FUP-clade scheme is derived directly from phylogenetic analysis of the conserved usher protein. This phylogenetic analyses also allows known and yet to be identified CFs to be analyzed in context with other CU adhesins present in other bacterial species which can aid in describing their evolutionary history.

A central advantage of genome-based analysis is that these serologically defined families can now be tested against usher-protein phylogeny: the CFA/I-like and CS5-like families fall within the α-clade, and the Class 1b group corresponds to the γ2 subgroup, confirming that the older antigenic classification largely reflects genuine evolutionary relationships.

## 3. Limitations of Current Detection of Colonization Factors

### 3.1. Conventional Approaches to CF Detection

Current approaches to detecting CFs in ETEC largely rely on targeted molecular and phenotypic assays. The most commonly used methods include conventional or multiplex polymerase chain reaction (PCR), immunological assays such as dot blot and enzyme-linked immunosorbent assay (ELISA), and more recently, high-throughput molecular platforms including TaqMan Array Cards (TACs); their relative strengths and limitations are summarized in Table 2.

However, PCR-based workflows are labor-intensive because they require prior bacterial culture and isolation of colonies before testing. Furthermore, due to the high number of known CFs in ETEC, PCR panels typically cover only a subset of CFs—e.g., CFA/I, CS1–CS8, CS12, CS14, CS17, CS19 and CS21 [46]. Being a genotypic method, PCR cannot report phenotypic expression, which varies with growth conditions; ELISA and dot blot are used to complement it, though both carry their own caveats— toxin ELISAs, for instance, are strongly affected by culture conditions, growth media, phase variation, and plasmid stability [47,48,49,50,51,52].

Dot blot remains the phenotypic gold standard for CF detection [47], but standardization between laboratories is difficult, monoclonal antibodies are often unavailable for rarer CFs, and interpretation of weak reactions can be subjective. Critically, a negative phenotypic result does not rule out the corresponding CF genes, since in vitro conditions may not mimic the gut; and loss of virulence plasmids during subculture can produce false negatives—a limitation shared with PCR, since both depend on cultured isolates [47,53].

TAC bridges PCR and genome-scale detection, targeting up to 18 CFs alongside toxin genes directly from stool, without culture [54,55]. Its main epidemiological limitation is that, applied to whole stool, it detects total virulence gene content rather than linking specific CF and toxin profiles to individual strains—a particular problem where mixed ETEC infections are common [55].

This is particularly problematic in settings where mixed ETEC infections are common, as multiple strains with distinct virulence profiles may coexist in the same host. Consequently, TAC cannot reliably determine specific CF combinations within individual isolates, which is key for understanding circulating strain profiles and informing vaccine development strategies.

Method standardization remains one of the greatest challenges in ETEC diagnostics and surveillance. Variability in sample collection, storage conditions, bacterial culture techniques, CF induction protocols, primer design, antibody availability, and interpretation thresholds can all influence detection outcomes. Differences between genotypic and phenotypic methods further complicate comparisons across studies. For example, PCR may detect CF genes that are not phenotypically expressed in vitro, whereas immunological assays may fail to detect CFs because of suboptimal induction conditions despite the genes being present. Similarly, direct molecular assays such as TAC may identify low-abundance targets of uncertain clinical significance.

**Figure 1:**
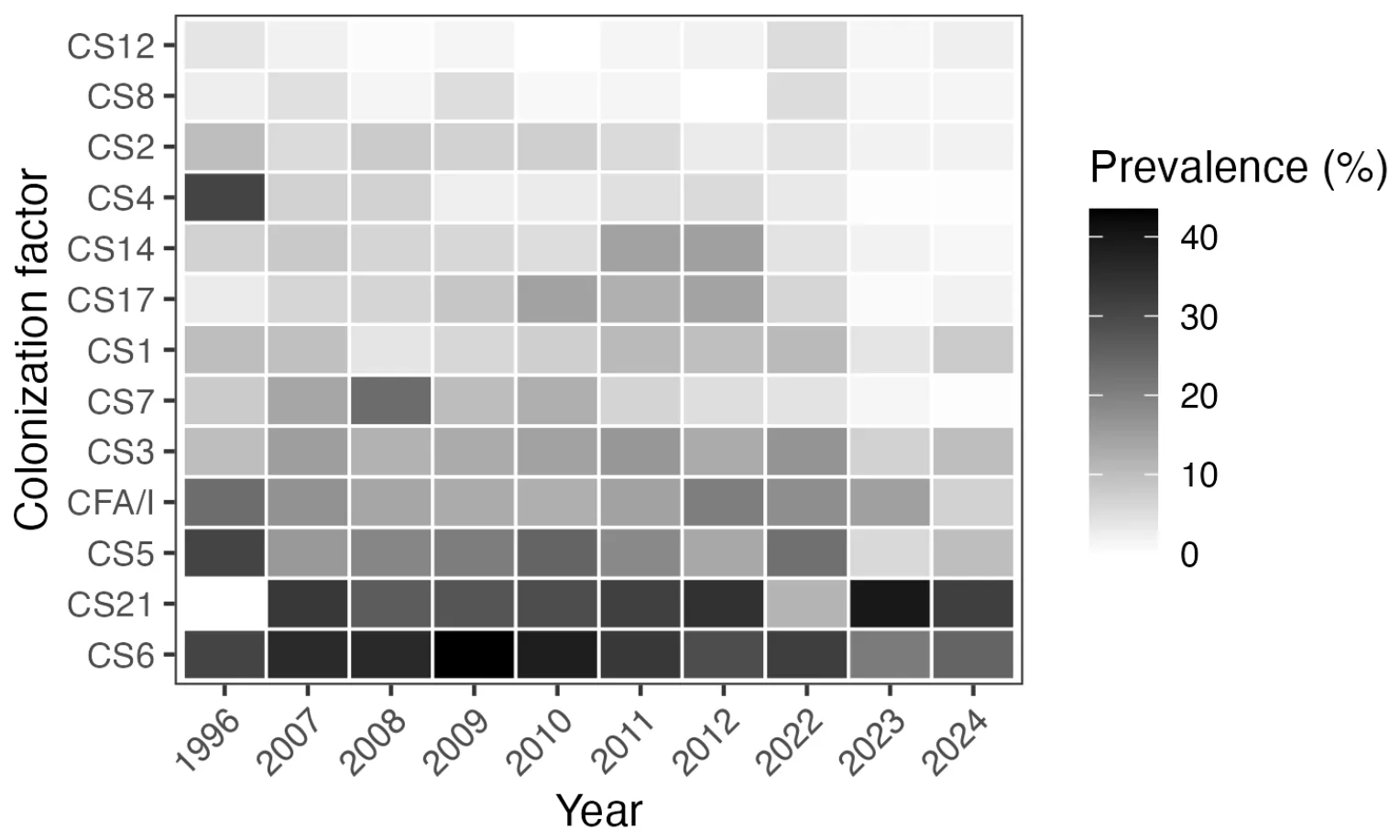
Prevalence of major colonization factors (CFs) among ETEC isolates collected through the 2% diarrheal surveillance system at icddr,b (Dhaka, Bangladesh), compiled from published PCR-based surveillance studies [21,56,57,58,59]. Only CFs included in the detection panels of all studies were incorporated into the analysis. For CFs reported as combinations (e.g., CS4+CS6 or CS5+CS6), prevalence was calculated based on the presence of each individual CF. The heatmap was generated in R. See the Data Availability Statement for further details.

To illustrate temporal changes in CF prevalence, we compiled published prevalence estimates from longitudinal surveillance studies performed through the 2% surveillance system at icddr,b in Dhaka, Bangladesh (Figure 1). Bangladesh represents one of the few settings where ETEC has been monitored over several decades using a relatively consistent surveillance framework, providing a valuable opportunity to examine changes in CF prevalence over time. To maximize comparability across studies, only CFs included in the PCR detection panels of all studies were incorporated into the analysis. Prevalence values were extracted directly from the original publications, and no attempt was made to recalculate prevalence estimates.

Although all prevalence estimates included in Figure 1 were based on PCR results, differences remained in study design, sampling strategies, laboratory protocols, and the composition of the study populations. Several studies also used dot blot assays to confirm PCR findings or characterize subsets of isolates; however, these data were not used when compiling the figure. Consequently, Figure 1 should be interpreted as illustrating overall temporal trends in reported CF prevalence rather than providing directly comparable prevalence estimates across studies.

Comparative analyses across countries and over longer time periods remain difficult because isolate-level data are rarely made publicly available—a problem we return to in Section 7.

### 3.2. Genomic Approaches to CF Classification

Advances in whole-genome sequencing (WGS) have changed our ability to characterize CFs in ETEC. Unlike targeted PCR-based approaches, genome-based analyses enable the detection of both known and previously unrecognized adhesin gene clusters. Recent studies have demonstrated that applying genomic approaches substantially increases CF detection rates and reveals a broader diversity of adhesins than previously appreciated. CS23 is a clear example of this, discussed in detail in Section 4.2.

More recently, representative collections of ETEC isolates from surveillance studies have been whole-genome sequenced, enabling genomic approaches to investigate CF prevalence and diversity. Several methods can be used to determine CF profiles from genomic data, including BLAST-based tools such as Abricate (https://github.com/tseemann/ABRICATE, accessed on 10 September 2026) or the k-mer-based tool Lexicmap (https://github.com/shenwei356/LexicMap, accessed on 10 September 2026) in combination with curated or manual databases. The most up-to-date database for ETEC virulence factors is the VFDB (https://www.mgc.ac.cn/VFs/, accessed on 10 September 2026), which has recently been expanded to include a comprehensive set of ETEC virulence factors through the ETECFinder database, which incorporates multiple alleles for both toxins and CFs [60]. Application of ETECFinder to more than 1000 publicly available genomes has revealed a continuum of variation across the CF genes, particularly within individual FUP clades. Notably, CF operons often contain genes that are highly similar to those found in multiple CF types within the same clade, especially chaperones and ushers and to some extent also minor subunits. As a result, sequence-based analyses may return best matches to different CF variants for individual genes within an operon, complicating classification. These patterns likely reflect underlying sequence similarity and diversification rather than true chimeric CFs, thereby making accurate CF profiling challenging [60].

A consistent observation across multiple surveillance studies is that a substantial proportion of ETEC isolates remain negative for CFs when screened using PCR panels targeting the most common CFs (CFA/I, CS1-CS8, CS12, CS14, CS17, CS19 and CS21). The proportion of CF-negative isolates varies considerably even within a single setting over time: the same Bangladeshi surveillance studies underlying Figure 1 have individually reported CF-negative rates ranging from 20% to 70% [21,56,57,58,59]; these are the same studies which Figure 1 is based on. These findings suggest that the true prevalence of CFs is underestimated in routine surveillance. Assessing prevalence is further complicated by the use of different detection methods across studies—most commonly PCR, which targets only a subset of CFs and therefore results in a high proportion of CF-negative isolates and a skewed picture of which CFs circulate. A more fundamental obstacle, discussed in Section 7, is that the underlying prevalence data are rarely reported in a form that permits comparison or pooling across studies.

Despite these advantages, accurate identification of CFs from genomic data is not straightforward. CF operons may be fragmented across contigs in draft genome assemblies, particularly when short-read sequencing is used. In addition, sequence variation within CF genes—including different alleles, and mosaic operon structures—can complicate detection when relying on strict sequence similarity thresholds. As a result, standard annotation pipelines may fail to identify divergent or partial CF loci, leading to continued underestimation of CF heterogeneity even in genomic datasets.

Integrating genomic data with epidemiological studies will provide a more accurate picture of circulating CFs and help inform the design of next-generation vaccines with broad ETEC protection.

Recent genomic analyses support this notion, revealing that many isolates classified as CF-negative based on PCR screening harbor gene clusters encoding either known but rarely screened CFs (e.g., CS23, CS30) or putative novel adhesins [61,62].

## 4. Emerging and Underdetected Colonization Factors

### 4.1. CS23: An Emerging and Previously Underdetected Colonization Factor

Among the emerging κ-class chaperone–usher fimbriae, CS23 has gained increasing attention due to its rising prevalence and broad distribution across ETEC populations [61,62]. The CF CS23 was first described in 2007 in the ETEC strain 1766a which was isolated from a child with diarrhea in 1985 in Santiago, Chile. ETEC 1766a, an O4 strain expressing both LT and ST, was shown to harbor no other ETEC CFs [63]. Among published ETEC surveillance studies since CS23 was described, CS23 has not been included in routine PCR panels and therefore the prevalence has been impossible to estimate.

In recent years, two independent studies originating from two geographically distinct locations, Bolivia and Bangladesh, have been conducted, revealing a more pronounced role of CS23-positive ETEC in diarrheal disease than previously appreciated. The authors of the Bolivian study collected routine samples between 2013 and 2014 from both patients with diarrhea and environmental water samples, and through sequencing and comparative genomics identified CS23 as the main CF in multiple clinical and environmental samples. The study further demonstrates that the CS23-positive isolates differ in their genetic background, i.e., they fall into distinct ETEC lineages, and identify multiple variants of the major subunit aalE compared to the reference CS23 aalE (strain 1766a). During 2022 and 2023, Dhaka, Bangladesh, experienced an upsurge of diarrheal cases due to a rise in diarrhea caused by *Vibrio cholerae* and ETEC [58]. A longitudinal study, including a global ETEC collection along with an unbiased selection of ETEC isolates, part of the 2% surveillance program at icddr,b, was initiated with the aim to try and understand the underlying genomic factors involved in the 2022–2023 outbreaks. A large subset of the isolates collected during the upsurge were, with comparative genomic analyses, shown to harbor an operon encoding CS23. Phylogenetic analyses showed a similar picture as in the Bolivian study, the CS23-positive strains fell into multiple distinct lineages. In both studies, it was suggested that the CS23 locus is plasmid-encoded and acquired independently of chromosomal background. These observations are consistent with a model in which CS23 spreads through plasmid dissemination and is not restricted to specific clonal lineages, which explains why CS23 is observed in diverse locations in the ETEC phylogeny. However, to study the roles of plasmids in the spread of CS23 would require complete circularized genomes. Interestingly, in the Bangladeshi study the CS23-positive ETEC were associated with severe disease in adults [61] and one possible explanation is that limited prior exposure to CS23-positive ETEC exposure resulted in lower levels of pre-existing immunity among adults. However, alternative explanations cannot be excluded, including differences in the virulence repertoire or genetic background of CS23-positive strains, or epidemiological factors associated with the outbreak.

The adherence properties of CS23 have been investigated in two independent studies, revealing that its contribution to epithelial attachment may be more complex than initially appreciated. While Del Canto et al. demonstrated that CS23 mediates adherence to Caco-2 cells in the prototype strain 1766a [63], Calderón-Toledo et al. showed that only a subset of CS23-positive clinical and environmental isolates from Bolivia adhered under the experimental conditions tested [62]. The discrepancies between these studies could be due to several factors. For example, variations in the CS23 protein sequence of the major subunit AalE which is considered the main structural component involved in adherence [63]. It may also be due to differences in the experimental setup, including how the bacteria were cultured, which affects the expression levels of CFs, for adherence assays. The diversity of epithelial cell models currently used for ETEC adherence assays—including Caco-2, HT-29, T84, HuTu80, and human enteroids—highlights the lack of standardized experimental approaches [30,64,65,66]. Given the diversity of CFs and their likely differences in receptor specificity, future studies may benefit from evaluating adherence across multiple intestinal cell models, both human and animal, under standardized growth conditions.

Taken together, CS23 illustrates how genome-based surveillance can distinguish between genuine epidemiological emergence and historical underdetection. Although important questions remain regarding its biology and contribution to virulence, current evidence indicates that CS23 is both an emerging and previously underdetected colonization factor whose epidemiological significance is only now becoming apparent.

### 4.2. γ-FUP Adhesins Expand the Known Colonization Factor Repertoire

In addition to CS23, several other less-characterized CFs belonging to the γ-class CU fimbriae—particularly within the γ2 subfamily—have been identified through genomic analyses, including CS26, CS27, CS28, and CS30. Similar patterns are as seen for CS23; these CFs are detected across multiple ETEC lineages and phylogenetic backgrounds, consistent with repeated acquisition through horizontal gene transfer rather than lineage-specific inheritance [67]. Early work using degenerate PCR targeting conserved regions of class 1b and related fimbriae revealed the presence of multiple previously unrecognized adhesin genes in isolates lacking known CFs, increasing CF detection rates by nearly 20% [68]. Subsequent genomic and functional studies have confirmed that CS26 and CS30 encode functional adhesins. For example, CS26 has been shown to mediate adherence to epithelial cells and shares structural and antigenic features with related γ2-class CFs [67], while CS30 is a plasmid-encoded CF associated with diarrheal disease and capable of mediating adhesion to intestinal epithelial cells [64,69]. Operons encoding CS27 and CS28 have only been described genomically; additional functional experiments to ascertain their role in adhesion are needed, for example, by showing that the genes are functional and responsible for the production of surface proteins conferring adhesion to human and/or animal cell lines.

Importantly, comparative genomic analyses have revealed that CF operons within the γ-class do not always conform to discrete, well-defined types. Instead, there appears to be a continuum of variation across genes encoding these adhesins, including allelic diversity within structural subunits, and the presence of mosaic gene clusters. This genetic plasticity likely reflects ongoing evolution and diversification of adhesins and may contribute to the difficulty in detecting and categorizing CFs using conventional PCR-based approaches. This challenges the traditional classification of CFs as discrete entities and instead supports a model of continuous diversification within CU fimbrial operons.

## 5. Animal Colonization Factors and Evolutionary Links

### 5.1. Major Animal CFs and Overlap with Human CFs

CFs (often referred to as F antigens) associated with animal ETEC—most prominently F4 (K88), F5 (K99), F6 (987P), F17, F18, and F41—are key virulence determinants in diarrheal disease in pigs, calves, and lambs. These fimbriae are largely found within the κ- and γ-class chaperone–usher (CU) families, which also include several CFs described in human ETEC isolates. This shared phylogenetic placement highlights an evolutionary link between CFs found in human and animal hosts.

**Figure 2:**
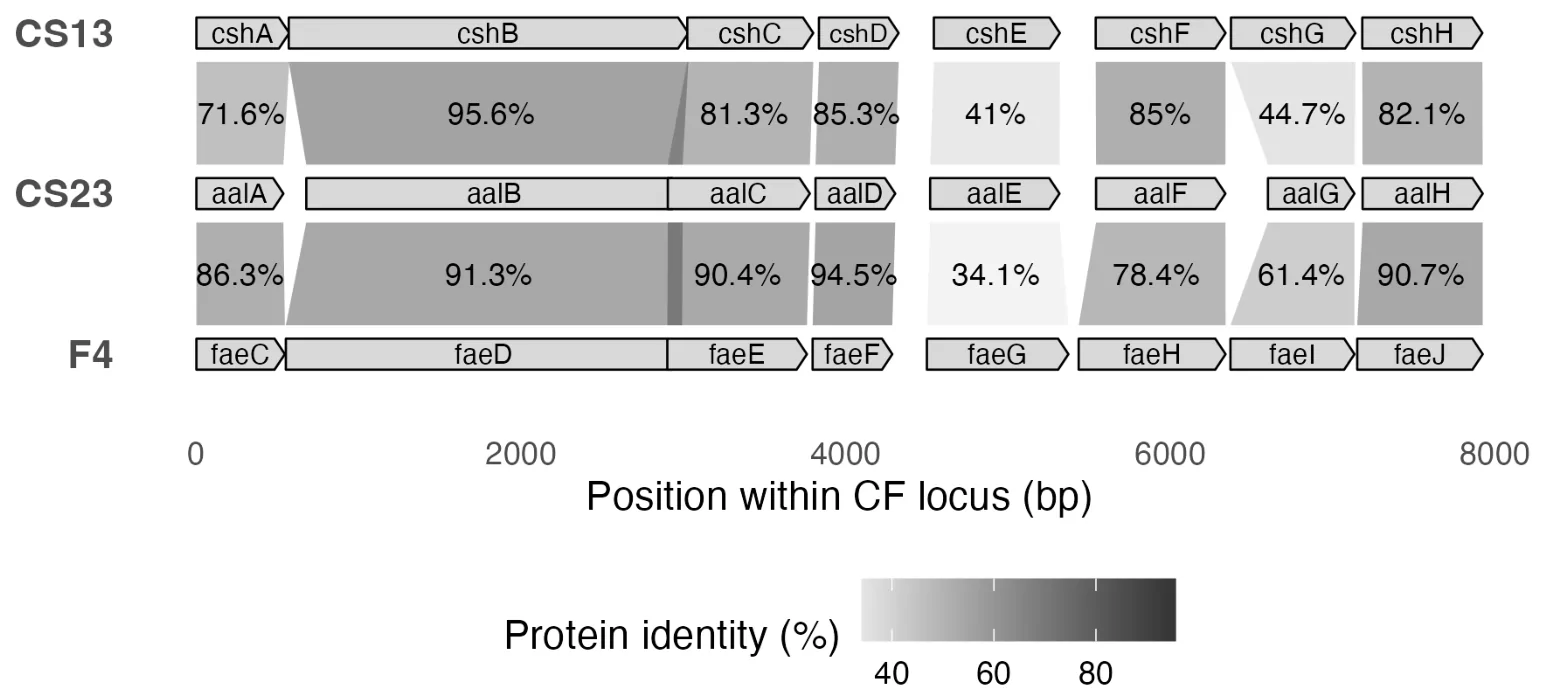
Comparative organization and protein sequence conservation of representative κ-FUP adhesin operons. Representative operons encoding the human colonization factors CS13 (GenBank accession OU964063) and CS23 (JQ434477), together with the porcine fimbrial operon F4/K88 (CP002730), are shown. Arrows represent annotated coding sequences and indicate gene orientation. Grey polygons connecting homologous genes illustrate pairwise amino acid sequence identity, with shading proportional to sequence identity (darker shading indicates higher identity). Percentage values indicate pairwise amino acid identity between orthologous proteins. The figure was generated using a custom R workflow that extracts annotated coding sequences from GenBank records, translates proteins, calculates pairwise global amino acid sequence identities, and visualizes operon organization using the gggenes (v0.7.0) package. See the Data Availability Statement for further details.

Several human-associated CFs show clear genetic relationships to animal-associated fimbriae. The genetic organization of the operon is conserved across members of the class 1b family and the γ2-FUP clade including CS12, CS18, CS20, and CS30 and the porcine fimbria F6 (987P). The κ-FUP clade encompasses both human and animal-associated CFs (CS13, CS23, F4, F5, and F18), where CS13, CS23 and F4 are encoded by highly similar operons, whereas the adhesin shares approximately 34–41% protein sequence identity (Figure 2). This pattern is consistent with a shared evolutionary origin and raises the possibility that related adhesins have diversified across different host-associated lineages.

### 5.2. Evolutionary Implications

Recent work has provided more direct evidence for the potential of CFs to mediate cross-host interactions. An atypical κ-class fimbria (F4-like) identified in a human ETEC strain was shown to mediate adhesion to epithelial cells from multiple hosts, including human, bovine, and porcine origins [34]. Functional assays demonstrated that this fimbria plays a dominant role in adhesion, suggesting that certain CFs may act as broad host-range adhesins rather than strictly host-specific determinants. Notably, this strain carried multiple CFs on a single plasmid, including homologs of both human- and animal-associated adhesins, highlighting the role of plasmids as platforms for the assembly and dissemination of diverse adhesion repertoires [34].

The presence of phylogenetically related CFs across hosts, combined with evidence of multi-host adhesion and plasmid-mediated transfer, raises the possibility that some ETEC lineages may have greater potential for cross-host dissemination than is currently recognized, although additional genomic and epidemiological data are required to test this hypothesis. This has important implications for understanding zoonotic transmission, the evolution of host specificity, and the design of broadly protective vaccines targeting conserved adhesin families. Detecting this cross-host movement requires looking for it: a human-centric panel screening only human-associated CFs cannot reveal an animal-associated adhesin in a human isolate, and vice versa, and the true prevalence of such events will remain unknown until studies screen beyond their host-specific CF set.

Together, these observations suggest that the distinction between human- and animal-associated colonization factors may be less absolute than traditionally assumed. However, evidence for cross-host dissemination of CFs remains limited, and its frequency, epidemiological importance, and contribution to ETEC evolution warrant further investigation.

## 6. Implications for ETEC Vaccine Design and Surveillance

The growing recognition of underdetected and emerging colonization factors (CFs) has important implications for both ETEC surveillance and vaccine development.

The selection of CFA/I and CS1–CS6 as the basis for current ETEC vaccine candidates [16,18,19,70,71,72,73] was a well-founded strategy: these CFs were, on the available evidence, the most prevalent and best characterized, and they remain so in many settings. Moreover, cross-reactivity between related CFs means that vaccine coverage may extend beyond the antigens directly included; oral inactivated candidates have induced immune responses against CFs not present in the formulation [74]. Whether this cross-reactivity extends to more recently described CFs, including members of the κ- and γ-FUP clades discussed in this review, remains unknown. Determining the breadth of cross-reactive immunity across increasingly divergent adhesins will therefore be important for evaluating both current vaccine coverage and the potential need for future antigen updates. None of this implies that these vaccines will fail. Rather, it argues for continued surveillance to track which CFs circulate over time, so that vaccine composition can stay ahead of a changing pathogen population rather than reflecting a single historical snapshot.

Similar challenges have been encountered in other bacterial vaccines targeting genetically diverse pathogens. Pneumococcal conjugate vaccines have progressively increased in valency to improve serotype coverage, while vaccine development against serogroup B Neisseria meningitidis has instead focused on identifying conserved protein antigens capable of providing broader protection across diverse strains [75,76,77,78].

ETEC vaccine development may ultimately face a similar strategic choice between continued expansion of CF antigen panels and targeting antigens that are more broadly distributed or conserved across ETEC populations or even can induce cross-reactive antibodies. EtpA and EatA represent the most extensively studied non-canonical ETEC vaccine antigens [79]. Both proteins are immunogenic during natural human infection and experimental challenge, while vaccination studies in animal models have demonstrated reduced intestinal colonization and suggest additive protective effects when the antigens are combined [80,81,82,83,84]. Nevertheless, their development remains at the preclinical stage, and it is currently unknown whether these antigens can provide protection comparable to or broader than existing CF-based vaccine strategies in humans. Therefore, rather than replacing CF-based vaccines, EtpA and EatA could act as complementary antigens that may expand protection against strains expressing diverse or previously unrecognized CFs.

In addition, integrating genomic surveillance into vaccine development pipelines will be critical for monitoring the emergence and spread of CFs over time. A more comprehensive understanding of CF diversity, distribution, and evolution will allow for more informed decisions regarding antigen selection and vaccine coverage.

## 7. Future Directions

A recurring theme of this review is that two things shape what we see as CF prevalence: real diversification of the adhesins themselves, and the limits of how we detect them. These are hard to tell apart. When a CF appears to rise, fall, or go missing, we rarely know how much is biology and how much is method. The clearest example is the 20–70% of isolates that screen CF-negative. Rather than treat that as a fixed property of ETEC, these isolates should be re-examined by sequencing, which in recent studies has reassigned many of them to rarely screened or previously unknown CFs [61,62].

### 7.1. CFs as a Continuum, Not a Fixed Set

CFs are more variable than their names suggest. Within a single CF there can be allelic differences in the subunits, mosaic operons, and plasmid- or lineage-linked variation, and this is most pronounced in the γ- and κ-clades. We need to stop treating CFs only as countable, fixed types and start treating them as points within a population of adhesins that is still diversifying [60]. Just as important is linking this variation to function—whether allelic and structural differences actually change how well a strain colonizes, what it binds, which hosts it infects, and how the immune system sees it.

This has a direct consequence for vaccines. If the circulating adhesins form a continuum rather than a fixed set, adding each new CF to a multivalent vaccine becomes impractical as the list grows. The alternative is to target what is shared across the diversity—non-fimbrial adhesins such as EtpA and EatA, which are immunogenic and protective in animal models [83,84], or conserved parts of the chaperone–usher machinery itself. These are attractive because they do not depend on first detecting and naming every variant. Whether they protect as well as the current CF-based vaccines is still an open question, and an important one. If cross-host dissemination proves to be more common than currently recognized, this could influence our understanding of ETEC reservoirs, dissemination pathways, and the long-term evolution of colonization factors. It may also have implications for surveillance priorities and future vaccine antigen selection.

### 7.2. The Isolate-Level Data Gap

Most of the above depends on data that are hard to get, and fixing this is probably the simplest high-impact change the field could make. The isolate-level data behind surveillance studies and vaccine trials are rarely published in a usable form. When we tried to compile CF prevalence across published studies for this review, only a handful reported the numbers in a way we could actually extract and analyze—most had them buried in the text or in figures rather than deposited as a simple table or CSV. As a result it is currently not possible to pool published data into a reliable global estimate of CF prevalence, even though that is exactly what vaccine coverage estimates rest on. This is a reporting problem, not a sequencing one. If raw, isolate-level data were deposited routinely, prevalence could be re-estimated as methods improve and new CFs are found, instead of being frozen at the moment each paper is published.

### 7.3. Towards Harmonized, Complementary Detection

Current prevalence estimates come from a mix of methods—PCR, dot blot, ELISA, TAC, and increasingly sequencing—that detect different things and respond differently to how samples are handled, cultured, and interpreted. This makes datasets hard to compare across regions or over time. As the list of known CFs grows and ETEC vaccines move forward, the field needs agreed protocols, reference databases, and typing schemes so that results from different labs can actually be put side by side.

Finally, it helps to be clear about what each method is for. PCR is fast, cheap, and standardized for known targets, but it reads the most variable parts of CF operons, so divergent alleles can slip through as false negatives, and it only tells you the presence or absence of known CFs. That is not a flaw—PCR was never meant to resolve evolutionary diversity. For questions about how ETEC adhesins are diversifying and moving between lineages and hosts, sequencing is the right tool: it picks up both known and divergent CFs, catches mosaic operons that do not fit the existing types, and helps decide which adhesins are worth following up functionally. Its main limitation now is access—sequencing is still not routine in many places where ETEC is common. Closing that gap, and feeding genomic surveillance into both monitoring and vaccine development, is what will give us an accurate and continuously updated picture of CF diversity.

Ultimately, integrating comparative genomics, standardized surveillance, functional characterization, and One Health sampling will be essential for building a more complete understanding of ETEC colonization factor diversity and ensuring that future diagnostics and vaccines keep pace with the continuing evolution of this pathogen.

## 8. Conclusions

CF diversity in ETEC is likely substantially underestimated, representing a major blind spot in current surveillance and vaccine development. Evidence from genomic studies reveals a broader and more dynamic landscape of adhesins than previously appreciated, including emerging and underdetected CFs distributed across diverse lineages and hosts. These findings challenge the traditional view of CFs as a limited and well-defined set of virulence determinants that are primarily disseminated through vertical transmission and instead point to an ongoing diversification driven by plasmid dissemination. This evolving picture does not diminish the value of current vaccine candidates, but it underscores why surveillance must continue alongside vaccine development, so that interventions can keep pace with the pathogen.

## Figures and Tables

**Table 1:** Structural classification of human ETEC colonization factors according to assembly mechanism, receptor-binding strategy, and representative CFs.

Structural Class	Assembly Mechanism	Receptor-Binding Strategy	Representative Human CF(s)
Rigid fimbriae	Alternate chaperone–usher (ACU)	Tip-localized minor-subunit adhesin confers glycan specificity (e.g., CfaE of CFA/I binds glycosphingolipids, asialo-GM1 and Lewis).	CFA/I, CS1, CS2, CS4, CS14, CS17, CS19 (Class 5 family and α-FUP) [15,36,37]
Fibrillar	Classical chaperone–usher (CU)	Thin, flexible filaments; receptor binding is mediated by the major structural subunit and/or terminal adhesin. Reported to recognise Sialic acid-containing glycoproteins; galactosylated glycans	CS3 (γ3) [38]
Afimbrial/non-fimbrial	Classical chaperone–usher (CU)	Polyvalent surface adhesin without a distinct rod. CS6 (CssA/CssB) binds sulfatide and fibronectin and exhibits broad adhesive properties.	CS6 (γ3) [39,40,41]
Double-helical fibrillar [*]	Alternate Chaperone–usher (ACU)	Host receptors remain poorly characterized. For CS7, weak binding to globotriaosylceramide and lactosylceramide has been reported.	CS5, CS7 (α-FUP subgroup [42])
Type IV pili	Type IV pilus machinery (prepilin peptidase, dedicated ATPase)	Retractile filaments mediating adhesion, microcolony formation and biofilm development; recognises sialic acids	CS8 (CFA/III), CS21 (Longus) [43,44]
Non-classical/non-CU adhesins	Two-partner secretion (EtpA); autotransporter/serine protease (EatA)	Direct host-cell binding independent of the CU pathway. EtpA recognizes blood-group glycans and bridges bacteria to host cells via the flagellar tip.	EtpA, EatA [32,45]

* Unlike the classical Class 5 fimbriae (e.g., CFA/I), individual CS5 filaments form thin, flexible fibrils that frequently intertwine into paired, double-helical structures as observed by electron microscopy.

**Table 2:** Comparison of methods currently used for detection and characterization of enterotoxigenic *Escherichia coli* (ETEC) colonization factors (CFs).

Method	Detects	Culture?	Applications	Strengths	Limitations
*Genotypic methods*					
Conventional/multiplex PCR	CF and toxin genes	Yes	Diagnostics; surveillance	Rapid; inexpensive; standardized; readily comparable between laboratories; new targets easily incorporated.	Limited to predefined targets; cultured isolates required; no information on expression or function.
TaqMan Array Card (TAC)	Multiple virulence genes	No	Clinical diagnostics; surveillance	Culture-independent; simultaneous detection of multiple pathogens and virulence genes; high sensitivity.	Cannot assign CFs and toxins to individual strains; mixed infections complicate interpretation; limited to included targets.
Whole-genome sequencing (WGS)	Whole-genome virulence content	Usually	Genomic surveillance; research	Detects known virulence genes; identifies allelic diversity; provides phylogenetic and genomic context; sequence data can be reanalyzed.	Higher cost; bioinformatic expertise required; predicts genetic potential rather than expression or function.
Genome-based CF prediction	Known CF genes and variants	–	Genome annotation; comparative genomics	Rapid screening of large genome collections; retrospective analyses; databases easily updated as new CFs are identified.	Dependent on reference databases and assembly quality; may miss divergent or novel CFs; predicts gene presence rather than expression or function.
Complete genome assembly	Complete genomes and plasmids	Yes	Plasmid biology; comparative genomics	Resolves complete CF operons, plasmids and structural variation; accurately determines genomic context.	Higher cost; lower throughput; currently unsuitable for routine surveillance.
*Phenotypic methods*					
Dot blot immunoassay	Surface CF proteins	Yes	CF expression; vaccine studies	Demonstrates CF expression; highly specific when antibodies are available; widely used phenotypic reference method.	Requires CF-inducing growth; antibody dependent; variable expression; limited to known CFs.
ELISA	Secreted toxins (± CF proteins)	Yes	Protein expression	Confirms protein production; relatively simple laboratory method.	Strongly influenced by growth conditions; limited use for CF detection; antibody dependent.
*Functional methods*					
Cell adherence assays	Cell adhesion	Yes	Functional studies; receptor identification	Demonstrates biological function; enables mutant comparisons; identifies receptor or cell specificity.	Poorly standardized; influenced by growth conditions, cell model and receptor availability; low throughput.
Animal models/organoid or enteroid systems *	Colonization and virulence	Yes	Mechanistic studies; vaccine evaluation	Most physiologically relevant assessment of colonization; useful for mechanistic and vaccine studies.	Few established models; technically demanding; expensive; ethical considerations; not standardized for routine use.

* Human intestinal enteroids and organoid-derived monolayers are increasingly used as physiologically relevant alternatives to conventional epithelial cell lines, although they are not yet widely adopted for routine characterization of ETEC colonization factors.

## Data Availability

Figure 1 was generated from prevalence data extracted from previously published studies. Figure 2 was generated using publicly available genome sequences; GenBank accession numbers are provided in the corresponding figure legend. The R notebooks, annotated reference sequences and scripts used to process the data and generate the figures are publicly available at: https://github.com/vonMentzerLab/MDPI_Microorganism_Review_Emerging_and_Underdetected_CFs, accessed on 10 September 2026.

## Abbreviations

The following abbreviations are used in this manuscript, listed in alphabetical order:

ACU	alternate chaperone–usher
AMR	antimicrobial resistance
CF	colonization factors
CU	chaperone–usher
ELISA	ensyme-linked immunosorbent assay
ETEC	Enterotoxigenic *Escherichia coli*
*E. coli*	*Escherichia coli*
LT	heat-labile toxin
FUP	fimbrial usher protein
PCR	polymerase chain reaction
ST	heat-stable toxin
TAC	TaqMan Array Card
TD	travelers’ diarrhea
WGS	whole-genome sequencing

## References

[1] Khalil I., Walker R., Porter C.K., Muhib F., Chilengi R., Cravioto A., Guerrant R., Svennerholm A.M., Qadri F., Baqar S. (2021). Enterotoxigenic *Escherichia coli* (ETEC) Vaccines: Priority Activities to Enable Product Development, Licensure, and Global Access. Vaccine.

[2] WHO Preferred Product Characteristics for Vaccines Against Enterotoxigenic *Escherichia coli*. https://www.who.int/publications/i/item/who-preferred-product-characteristics-for-vaccines-against-enterotoxigenic-escherichia-coli.

[3] Mwendera C.A., Yilma M., Wairimu C., Kering K., Mwinjiwa E., Kariuki J.N., Asrat D., Mekasha A., Msefula C., Kariuki S. (2026). Burden of *Shigella* and Enterotoxigenic *Escherichia coli* Infections among Children under 5 Years in Ethiopia, Kenya and Malawi: A Systematic Review and Meta-Analysis. BMJ Glob. Health.

[4] Leung A.K., Leung A.A., Wong A.H., Hon K.L. (2019). Travelers’ Diarrhea: A Clinical Review. Recent Pat. Inflamm. Allergy Drug Discov..

[5] Lääveri T., Pakkanen S.H., Antikainen J., Riutta J., Mero S., Kirveskari J., Kantele A. (2014). High Number of Diarrhoeal Co-Infections in Travellers to Benin, West Africa. BMC Infect. Dis..

[6] Anderson J.D., Bagamian K.H., Muhib F., Amaya M.P., Laytner L.A., Wierzba T., Rheingans R. (2019). Burden of Enterotoxigenic *Escherichia coli* and *Shigella* Non-Fatal Diarrhoeal Infections in 79 Low-Income and Lower Middle-Income Countries: A Modelling Analysis. Lancet Glob. Health.

[7] Dubreuil J.D., Isaacson R.E., Schifferli D.M. (2016). Animal Enterotoxigenic *Escherichia coli*. EcoSal Plus.

[8] Fuad M., Mahmud Z., Mishu I.D., Tamanna S., Ansari F., Manik M.R.K., Hossain Howlader M.Z. (2026). Molecular Characterization of Extended-Spectrum *β*-Lactamase (ESBL) and Virulent Genes in Multidrug-Resistant *Escherichia coli* Isolated from Pharmaceutical and Environmental Wastewaters in Dhaka, Bangladesh. Sci. Rep..

[9] Xiang Y., Wu F., Chai Y., Xu X., Yang L., Tian S., Zhang H., Li Y., Yang C., Liu H. (2020). A New Plasmid Carrying mphA Causes Prevalence of Azithromycin Resistance in Enterotoxigenic *Escherichia coli* Serogroup O6. BMC Microbiol..

[10] Johura F.T., Sultana M., Sadique A., Monira S., Sack D.A., Sack R.B., Alam M., Chakraborty S. (2024). The Antimicrobial Resistance of Enterotoxigenic *Escherichia coli* from Diarrheal Patients and the Environment in Two Geographically Distinct Rural Areas in Bangladesh over the Years. Microorganisms.

[11] García A., Fox J.G. (2021). A One Health Perspective for Defining and Deciphering *Escherichia coli* Pathogenic Potential in Multiple Hosts. Comp. Med..

[12] Collignon P.J., McEwen S.A. (2019). One Health—Its Importance in Helping to Better Control Antimicrobial Resistance. Trop. Med. Infect. Dis..

[13] Zhang X., Li D., Apley M.D., Karriker L., Connor J.F., Bromfield C., Gebhardt J.T., Lubbers B., Kittana H., Pendell D. (2025). Challenges and Opportunities of Bacterial Vaccines as Alternatives to Antimicrobials in Swine Health Management: Insights from U.S. Veterinarians. Pathogens.

[14] Begum Y.A., Talukder K.A., Azmi I.J., Shahnaij M., Sheikh A., Sharmin S., Svennerholm A.M., Qadri F. (2016). Resistance Pattern and Molecular Characterization of Enterotoxigenic *Escherichia coli* (ETEC) Strains Isolated in Bangladesh. PLoS ONE.

[15] von Mentzer A., Svennerholm A.M. (2024). Colonization Factors of Human and Animal-Specific Enterotoxigenic *Escherichia coli* (ETEC). Trends Microbiol..

[16] Hossain M.J., Secka F., Sanyang L.C., Taiwo R., Okoh E.C., Olubiyi O.A., Drammeh M., Richard E.U., Balami A.D., Drammeh M. (2026). Efficacy of ETVAX, a Vaccine against Enterotoxigenic *Escherichia coli*-Positive Diarrhoea in Gambian Children: A Double-Blind, Randomised, Placebo-Controlled, Phase 2b Trial. Lancet Infect. Dis..

[17] Girardi P., Harutyunyan S., Neuhauser I., Glaninger K., Korda O., Nagy G., Nagy E., Szijártó V., Pall D., Szarka K. (2022). Evaluation of the Safety, Tolerability and Immunogenicity of ShigETEC, an Oral Live Attenuated *Shigella*-ETEC Vaccine in Placebo-Controlled Randomized Phase 1 Trial. Vaccines.

[18] Harro C., Bourgeois A.L., Sack D., Walker R., DeNearing B., Brubaker J., Maier N., Fix A., Dally L., Chakraborty S. (2019). Live Attenuated Enterotoxigenic *Escherichia coli* (ETEC) Vaccine with dmLT Adjuvant Protects Human Volunteers against Virulent Experimental ETEC Challenge. Vaccine.

[19] Upadhyay I., Parvej S.M.D., Li S., Lauder K.L., Shen Y., Zhang W. (2023). Polyvalent Protein Adhesin MEFA-II Induces Functional Antibodies against Enterotoxigenic *Escherichia coli* (ETEC) Adhesins CS7, CS12, CS14, CS17, and CS21 and Heat-Stable Toxin (STa). Appl. Environ. Microbiol..

[20] Vidal R.M., Muhsen K., Tennant S.M., Svennerholm A.M., Sow S.O., Sur D., Zaidi A.K.M., Faruque A.S.G., Saha D., Adegbola R. (2019). Colonization Factors among Enterotoxigenic *Escherichia coli* Isolates from Children with Moderate-to-Severe Diarrhea and from Matched Controls in the Global Enteric Multicenter Study (GEMS). PLoS Neglected Trop. Dis..

[21] Amin M.A., Akhtar M., Afrad M.H., Rahman S.I.A., Parvin N., Akter A., Tauheed I., Amin M.A., Ryan E.T., Khan A.I. (2025). Evaluation of Colonization Factors of ETEC Isolated from Diarrheal Patients in Bangladesh: A Comparative Approach Using Multiplex PCR and Dot Blot Immunoassay. J. Microbiol. Methods.

[22] von Mentzer A., Connor T.R., Wieler L.H., Semmler T., Iguchi A., Thomson N.R., Rasko D.A., Joffre E., Corander J., Pickard D. (2014). Identification of Enterotoxigenic *Escherichia coli* (ETEC) Clades with Long-Term Global Distribution. Nat. Genet..

[23] Qadri F., Khan A.I., Faruque A.S.G., Begum Y.A., Chowdhury F., Nair G.B., Salam M.A., Sack D.A., Svennerholm A.M. (2005). Enterotoxigenic *Escherichia coli* and *Vibrio cholerae* Diarrhea, Bangladesh, 2004. Emerg. Infect. Dis..

[24] Moon J., Barry E.M. (2021). Sequence Variations in the ETEC CS6 Operon Affect Transcript and Protein Expression. Virulence.

[25] Nagy B., Fekete P.Z. (2005). Enterotoxigenic *Escherichia coli* in Veterinary Medicine. Int. J. Med. Microbiol..

[26] Fleckenstein J.M., Sheikh A. (2021). Emerging Themes in the Molecular Pathogenesis of Enterotoxigenic *Escherichia coli*. J. Infect. Dis..

[27] Sabui S., Ghosal A., Dutta S., Ghosh A., Ramamurthy T., Nataro J.P., Hamabata T., Chatterjee N.S. (2010). Allelic Variation in Colonization Factor CS6 of Enterotoxigenic *Escherichia coli* Isolated from Patients with Acute Diarrhoea and Controls. J. Med. Microbiol..

[28] Hazen T.H., Nagaraj S., Sen S., Permala-Booth J., Canto F.D., Vidal R., Barry E.M., Bitoun J.P., Chen W.H., Tennant S.M. (2019). Genome and Functional Characterization of Colonization Factor Antigen I- and CS6-Encoding Heat-Stable Enterotoxin-Only Enterotoxigenic *Escherichia coli* Reveals Lineage and Geographic Variation. mSystems.

[29] Nuccio S.P., Bäumler A.J. (2007). Evolution of the Chaperone/Usher Assembly Pathway: Fimbrial Classification Goes Greek. Microbiol. Mol. Biol. Rev. MMBR.

[30] Cruz-Córdova A., Espinosa-Mazariego K., Ochoa S.A., Saldaña Z., Rodea G.E., Cázares-Domínguez V., Rodríguez-Ramírez V., Eslava-Campos C.A., Navarro-Ocaña A., Arrellano-Galindo J. (2014). CS21 Positive Multidrug-Resistant ETEC Clinical Isolates from Children with Diarrhea Are Associated with Self-Aggregation, and Adherence. Front. Microbiol..

[31] Patel S.K., Dotson J., Allen K.P., Fleckenstein J.M. (2004). Identification and Molecular Characterization of EatA, an Autotransporter Protein of Enterotoxigenic *Escherichia coli*. Infect. Immun..

[32] Roy K., Hilliard G.M., Hamilton D.J., Luo J., Ostmann M.M., Fleckenstein J.M. (2009). Enterotoxigenic *Escherichia coli* EtpA Mediates Adhesion between Flagella and Host Cells. Nature.

[33] Sjöling A., von Mentzer A., Svennerholm A.M. (2015). Implications of Enterotoxigenic *Escherichia coli* Genomics for Vaccine Development. Expert Rev. Vaccines.

[34] Inoue H., Tanimoto Y., Zheng D., Ban-Furukawa E., Inoue M., Omori Y., Yamaguchi Y., Tachibana T., Aso H., Zhang W. (2025). An Atypical Kappa-Class Chaperone-Usher Fimbriae of a Human Enterotoxigenic *Escherichia coli* Strain Shows Multi-Host Adherence and Distinct Phylogenetic Feature. Microbiol. Immunol..

[35] Del Canto F., O’Ryan M., Pardo M., Torres A., Gutiérrez D., Cádiz L., Valdés R., Mansilla A., Martínez R., Hernández D. (2017). Chaperone-Usher Pili Loci of Colonization Factor-Negative Human Enterotoxigenic *Escherichia coli*. Front. Cell. Infect. Microbiol..

[36] Zheng W., Andersson M., Mortezaei N., Bullitt E., Egelman E. (2019). Cryo-EM Structure of the CFA/I Pilus Rod. IUCrJ.

[37] Li Y.F., Poole S., Nishio K., Jang K., Rasulova F., McVeigh A., Savarino S.J., Xia D., Bullitt E. (2009). Structure of CFA/I Fimbriae from Enterotoxigenic *Escherichia coli*. Proc. Natl. Acad. Sci. USA.

[38] Wennerås C., Neeser J.R., Svennerholm A.M. (1995). Binding of the Fibrillar CS3 Adhesin of Enterotoxigenic *Escherichia coli* to Rabbit Intestinal Glycoproteins Is Competitively Prevented by GalNAc Beta 1-4Gal-containing Glycoconjugates. Infect. Immun..

[39] Lüdi S., Frey J., Favre D., Stoffel M.H. (2006). Assessing the Expression of Enterotoxigenic *Escherichia coli*-Specific Surface Antigens in Recombinant Strains by Transmission Electron Microscopy and Immunolabeling. J. Histochem. Cytochem. Off. J. Histochem. Soc..

[40] Jansson L., Tobias J., Jarefjäll C., Lebens M., Svennerholm A.M., Teneberg S. (2009). Sulfatide Recognition by Colonization Factor Antigen CS6 from Enterotoxigenic *Escherichia coli*. PLoS ONE.

[41] Roy S.P., Rahman M.M., Yu X.D., Tuittila M., Knight S.D., Zavialov A.V. (2012). Crystal Structure of Enterotoxigenic *Escherichia coli* Colonization Factor CS6 Reveals a Novel Type of Functional Assembly. Mol. Microbiol..

[42] Jansson L., Tobias J., Lebens M., Svennerholm A.M., Teneberg S. (2006). The Major Subunit, CfaB, of Colonization Factor Antigen I from Enterotoxigenic *Escherichia coli* Is a Glycosphingolipid Binding Protein. Infect. Immun..

[43] Sjöberg P.O., Lindahl M., Porath J., Wadström T. (1988). Purification and Characterization of CS2, a Sialic Acid-Specific Haemagglutinin of Enterotoxigenic *Escherichia coli*. Biochem. J..

[44] Giron J.A., Levine M.M., Kaper J.B. (1994). Longus: A Long Pilus Ultrastructure Produced by Human Enterotoxigenic *Escherichia coli*. Mol. Microbiol..

[45] Fleckenstein J.M., Lindler L.E., Elsinghorst E.A., Dale J.B. (2000). Identification of a Gene within a Pathogenicity Island of Enterotoxigenic *Escherichia coli* H10407 Required for Maximal Secretion of the Heat-Labile Enterotoxin. Infect. Immun..

[46] Rodas C., Iniguez V., Qadri F., Wiklund G., Svennerholm A.M., Sjöling Å. (2009). Development of Multiplex PCR Assays for Detection of Enterotoxigenic *Escherichia coli* Colonization Factors and Toxins. J. Clin. Microbiol..

[47] Sjöling Å., Wiklund G., Savarino S.J., Cohen D.I., Svennerholm A.M. (2007). Comparative Analyses of Phenotypic and Genotypic Methods for Detection of Enterotoxigenic *Escherichia coli* Toxins and Colonization Factors. J. Clin. Microbiol..

[48] Gonzales L., Ali Z.B., Nygren E., Wang Z., Karlsson S., Zhu B., Quiding-Järbrink M., Sjöling A. (2013). Alkaline pH Is a Signal for Optimal Production and Secretion of the Heat Labile Toxin, LT in Enterotoxigenic *Escherichia coli* (ETEC). PLoS ONE.

[49] Joffre E., von Mentzer A., Ghany M.A.E., Oezguen N., Savidge T., Dougan G., Svennerholm A.M., Sjöling A. (2015). Allele Variants of Enterotoxigenic *Escherichia coli* Heat-Labile Toxin Are Globally Transmitted and Associated with Colonization Factors. J. Bacteriol..

[50] Joffré E., von Mentzer A., Svennerholm A.M., Sjöling Å. (2016). Identification of New Heat-Stable (STa) Enterotoxin Allele Variants Produced by Human Enterotoxigenic *Escherichia coli* (ETEC). Int. J. Med. Microbiol..

[51] Crofts A.A., Giovanetti S.M., Rubin E.J., Poly F.M., Gutierrez R.L., Talaat K.R., Porter C.K., Riddle M.S., DeNearing B., Brubaker J. (2018). Enterotoxigenic *E. coli* Virulence Gene Regulation in Human Infections. Proc. Natl. Acad. Sci. USA.

[52] Tobias J., von Mentzer A., Frykberg P.L., Aslett M., Page A.J., Sjöling A., Svennerholm A.M. (2016). Stability of the Encoding Plasmids and Surface Expression of CS6 Differs in Enterotoxigenic *Escherichia coli* (ETEC) Encoding Different Heat-Stable (ST) Enterotoxins (STh and STp). PLoS ONE.

[53] Fairbrother J.M., Nadeau É. (2019). Colibacillosis. Diseases of Swine.

[54] Lappan R., Henry R., Chown S.L., Luby S.P., Higginson E.E., Bata L., Jirapanjawat T., Schang C., Openshaw J.J., O’Toole J. (2021). Monitoring of Diverse Enteric Pathogens across Environmental and Host Reservoirs with TaqMan Array Cards and Standard qPCR: A Methodological Comparison Study. Lancet Planet. Health.

[55] Liu J., Jokiranta T.S., Carlin N., Stroup S., Zhang J., Sjostrand B., Svennerholm A.M., Houpt E.R., Kantele A. (2025). Use of a TaqMan Array Card for Identification of Enterotoxins and Colonization Factors Directly from Stool Samples in an Enterotoxigenic *E. coli* Vaccine Study. Microbiol. Spectr..

[56] Qadri F., Das S.K., Faruque A.S.G., Fuchs G.J., Albert M.J., Sack R.B., Svennerholm A.M. (2000). Prevalence of Toxin Types and Colonization Factors in Enterotoxigenic *Escherichia coli* Isolated during a 2-Year Period from Diarrheal Patients in Bangladesh. J. Clin. Microbiol..

[57] Begum Y.A., Baby N.I., Faruque A.S.G., Jahan N., Cravioto A., Svennerholm A.M., Qadri F. (2014). Shift in Phenotypic Characteristics of Enterotoxigenic *Escherichia coli* (ETEC) Isolated from Diarrheal Patients in Bangladesh. PLoS Neglected Trop. Dis..

[58] Tauheed I., Ahmed T., Akter A., Firoj M.G., Ahmmed F., Rahman S.I.A., Afrad M.H., Islam M.N., Rahman A., Khan A.I. (2023). A Snap-Shot of a Diarrheal Epidemic in Dhaka Due to Enterotoxigenic *Escherichia coli* and *Vibrio cholerae* O1 in 2022. Front. Public Health.

[59] Harris A.M., Chowdhury F., Begum Y.A., Khan A.I., Faruque A.S.G., Svennerholm A.M., Harris J.B., Ryan E.T., Cravioto A., Calderwood S.B. (2008). Shifting Prevalence of Major Diarrheal Pathogens in Patients Seeking Hospital Care during Floods in 1998, 2004, and 2007 in Dhaka, Bangladesh. Am. J. Trop. Med. Hyg..

[60] Scheutz F., Nielsen C.H., von Mentzer A. (2024). Construction of the ETECFinder Database for the Characterization of Enterotoxigenic *Escherichia coli* (ETEC) and Revision of the VirulenceFinder Web Tool at the CGE Website. J. Clin. Microbiol..

[61] Rahman S.I.A., Jubair M., Akhtar M., Akter A., Tauheed I., Begum Y.A., Bhattacharjee P., Afrad M.H., Khanam F., Islam M.T. (2025). Genomic Insights into a Diarrheal Outbreak in Bangladesh Reveal Novel ETEC Lineages and Expansion of CS23 Colonization Factor. Microbiol. Spectr..

[62] Calderon Toledo C., von Mentzer A., Agramont J., Thorell K., Zhou Y., Szabó M., Colque P., Kuhn I., Gutiérrez-Cortez S., Joffré E. (2023). Circulation of Enterotoxigenic *Escherichia coli* (ETEC) Isolates Expressing CS23 from the Environment to Clinical Settings. mSystems.

[63] Del Canto F., Botkin D.J., Valenzuela P., Popov V., Ruiz-Perez F., Nataro J.P., Levine M.M., Stine O.C., Pop M., Torres A.G. (2012). Identification of Coli Surface Antigen 23, a Novel Adhesin of Enterotoxigenic *Escherichia coli*. Infect. Immun..

[64] von Mentzer A., Tobias J., Wiklund G., Nordqvist S., Aslett M., Dougan G., Sjöling Å., Svennerholm A.M. (2017). Identification and Characterization of the Novel Colonization Factor CS30 Based on Whole Genome Sequencing in Enterotoxigenic *Escherichia coli* (ETEC). Sci. Rep..

[65] Moal V.L.L., Servin A.L. (2013). Pathogenesis of Human Enterovirulent Bacteria: Lessons from Cultured, Fully Differentiated Human Colon Cancer Cell Lines. Microbiol. Mol. Biol. Rev. MMBR.

[66] Smith E.M., Grassel C.L., Papadimas A., Foulke-Abel J., Barry E.M. (2022). The Role of CFA/I in Adherence and Toxin Delivery by ETEC Expressing Multiple Colonization Factors in the Human Enteroid Model. PLoS Neglected Trop. Dis..

[67] Cádiz L., Torres A., Valdés R., Vera G., Gutiérrez D., Levine M.M., Montero D.A., O’Ryan M., Rasko D.A., Stine O.C. (2018). Coli Surface Antigen 26 Acts as an Adherence Determinant of Enterotoxigenic *Escherichia coli* and Is Cross-Recognized by Anti-CS20 Antibodies. Front. Microbiol..

[68] Nada R.A., Shaheen H.I., Khalil S.B., Mansour A., El-Sayed N., Touni I., Weiner M., Armstrong A.W., Klena J.D. (2011). Discovery and Phylogenetic Analysis of Novel Members of Class b Enterotoxigenic *Escherichia coli* Adhesive Fimbriae. J. Clin. Microbiol..

[69] Von Mentzer A., Zalem D., Chrienova Z., Teneberg S. (2020). Colonization Factor CS30 from Enterotoxigenic *Escherichia coli* Binds to Sulfatide in Human and Porcine Small Intestine. Virulence.

[70] Akhtar M., Chowdhury M.I., Bhuiyan T.R., Kaim J., Ahmed T., Rafique T.A., Khan A., Rahman S.I.A., Khanam F., Begum Y.A. (2019). Evaluation of the Safety and Immunogenicity of the Oral Inactivated Multivalent Enterotoxigenic *Escherichia coli* Vaccine ETVAX in Bangladeshi Adults in a Double-Blind, Randomized, Placebo-Controlled Phase I Trial Using Electrochemiluminescence and ELISA Assays for Immunogenicity Analyses. Vaccine.

[71] Qadri F., Akhtar M., Bhuiyan T.R., Chowdhury M.I., Ahmed T., Rafique T.A., Khan A., Rahman S.I.A., Khanam F., Lundgren A. (2020). Safety and Immunogenicity of the Oral, Inactivated, Enterotoxigenic *Escherichia coli* Vaccine ETVAX in Bangladeshi Children and Infants: A Double-Blind, Randomised, Placebo-Controlled Phase 1/2 Trial. Lancet Infect. Dis..

[72] Riddle M.S., Maciel M., Porter C.K., Poole S.T., Gutierrez R.L., Gormley R., Laird R.M., Sebeny P.J., Dori K.E., Greenleaf M.E. (2020). A First in Human Clinical Trial Assessing the Safety and Immunogenicity of Transcutaneously Delivered Enterotoxigenic *Escherichia coli* Fimbrial Tip Adhesin with Heat-Labile Enterotoxin with Mutation R192G. Vaccine.

[73] Poncet D., Hessler C., Liang H., Gautheron S., Sergent M., Rintala N.D., Seydoux E., Huang P.W.D., Argilla D., Ruiz S. (2020). Preclinical Optimization of an Enterotoxigenic *Escherichia coli* Adjuvanted Subunit Vaccine Using Response Surface Design of Experiments. npj Vaccines.

[74] Leach S., Lundgren A., Carlin N., Löfstrand M., Svennerholm A.M. (2017). Cross-Reactivity and Avidity of Antibody Responses Induced in Humans by the Oral Inactivated Multivalent enterotoxigenic *Escherichia coli* (ETEC) Vaccine ETVAX. Vaccine.

[75] Croucher N.J., Harris S.R., Fraser C., Quail M.A., Burton J., van der Linden M., McGee L., von Gottberg A., Song J.H., Ko K.S. (2011). Rapid Pneumococcal Evolution in Response to Clinical Interventions. Science.

[76] Acha Alarcon L., Seguí G., Piñeiro-Iglesias B., Svetlicic E., Kondori N., Gomila M., Moore E.R.B., Karlsson R. (2025). Identification of *Streptococcus pneumoniae*-Specific Proteins by Surface-Shaving Proteomics. J. Proteome Res..

[77] Seib K.L., Zhao X., Rappuoli R. (2012). Developing Vaccines in the Era of Genomics: A Decade of Reverse Vaccinology. Clin. Microbiol. Infect..

[78] Tan L.K.K., Carlone G.M., Borrow R. (2010). Advances in the Development of Vaccines against *Neisseria meningitidis*. N. Engl. J. Med..

[79] Kuhlmann F.M., Martin J., Hazen T.H., Vickers T.J., Pashos M., Okhuysen P.C., Gómez-Duarte O.G., Cebelinski E., Boxrud D., Canto F.D. (2019). Conservation and Global Distribution of Non-Canonical Antigens in Enterotoxigenic *Escherichia coli*. PLoS Neglected Trop. Dis..

[80] Kumar P., Luo Q., Vickers T.J., Sheikh A., Lewis W.G., Fleckenstein J.M. (2014). EatA, an Immunogenic Protective Antigen of Enterotoxigenic *Escherichia coli*, Degrades Intestinal Mucin. Infect. Immun..

[81] Buckley D.P., Akhtar M., Thapa M., Schmitz A., Turner J., Vickers T.J., Khatoon N., Kaisar M.H., Coggin J.A., Ganguli D. (2026). Human Enterotoxigenic *Escherichia coli* (ETEC) Infections Elicit Antibodies That Broadly Neutralize Mucinases of Pathogenic *Escherichia coli* and *Shigella*. Proc. Natl. Acad. Sci. USA.

[82] Fleckenstein J., Sheikh A., Qadri F. (2014). Novel Antigens for Enterotoxigenic *Escherichia coli* Vaccines. Expert Rev. Vaccines.

[83] Roy K., Hamilton D., Ostmann M.M., Fleckenstein J.M. (2009). Vaccination with EtpA Glycoprotein or Flagellin Protects against Colonization with Enterotoxigenic *Escherichia coli* in a Murine Model. Vaccine.

[84] Luo Q., Vickers T.J., Fleckenstein J.M. (2016). Immunogenicity and Protective Efficacy against Enterotoxigenic *Escherichia coli* Colonization Following Intradermal, Sublingual, or Oral Vaccination with EtpA Adhesin. Clin. Vaccine Immunol. CVI.

